# From Modified Haller Index to a Novel Patented Anatomical Measurement Device: Engineering Development, Validation, and Clinical Applications of Non-Invasive Thoracic Morphometry

**DOI:** 10.3390/bioengineering13070839

**Published:** 2026-07-21

**Authors:** Andrea Sonaglioni, Gian Luigi Nicolosi, Massimo Baravelli, Michele Lombardo

**Affiliations:** 1 Division of Cardiology, IRCCS MultiMedica, 20123 Milan, Italy; massimo.baravelli@multimedica.it (M.B.); michele.lombardo@multimedica.it (M.L.); 2 Division of Cardiology, Policlinico San Giorgio, 33170 Pordenone, Italy; gianluigi.nicolosi@gmail.com

**Keywords:** modified Haller index, thoracic morphometry, pectus excavatum, chest wall deformity, cardiovascular phenotyping, myocardial strain, stress echocardiography, pulmonary function, biomedical engineering, medical device

## Abstract

Thoracic morphology is increasingly recognized as an important determinant of cardiopulmonary phenotype, influencing cardiovascular mechanics, respiratory physiology, and the interpretation of diagnostic imaging findings. Although the radiological Haller Index (HI) remains the reference standard for quantifying pectus excavatum severity, its dependence on computed tomography and ionizing radiation limits widespread clinical implementation, particularly in settings requiring serial evaluations. To overcome these limitations, the Modified Haller Index (MHI) was developed as a simple, non-invasive, radiation-free alternative that combines external thoracic anthropometry with echocardiographic assessment. Since its introduction, the MHI has undergone clinical validation and has progressively expanded beyond the assessment of chest wall deformities, demonstrating that thoracic conformation is not merely an anatomical characteristic but a clinically relevant determinant of cardiovascular and respiratory physiology. Growing evidence indicates that thoracic morphology influences cardiac chamber geometry, ventricular filling, stroke volume, myocardial deformation, ventricular–arterial coupling, exercise stress echocardiography findings, pulmonary function, and symptom perception across a broad spectrum of cardiovascular and respiratory diseases. Elevated MHI values identify individuals with a reduced antero-posterior thoracic diameter and a distinctive cardiopulmonary phenotype characterized by external cardiac compression, smaller cardiac chambers, restrictive ventilatory physiology, and apparent alterations in myocardial mechanics despite the absence of intrinsic myocardial disease. Building upon the clinical validation of the MHI, a novel patented anatomical measurement device was engineered to standardize thoracic morphometric assessment by enabling direct acquisition of both latero-lateral and antero-posterior thoracic diameters within a single measurement procedure. The device integrates dedicated anatomical reference elements, an innovative adjustable sternal pointer, movable measurement components, and a standardized acquisition workflow into a portable, low-cost, and radiation-free platform, thereby improving measurement reproducibility while simplifying bedside MHI determination. This narrative review summarizes the historical evolution of thoracic morphometry, the development and clinical validation of the MHI, the engineering rationale, structural architecture, and measurement workflow of the patented device, and the growing evidence supporting the clinical significance of thoracic conformation across cardiovascular and respiratory medicine. Together, the MHI and the proposed anatomical measurement device establish a practical platform for standardized, radiation-free thoracic morphometry that may facilitate routine bedside phenotyping. Future integration with digital technologies, artificial intelligence, and advanced imaging systems may further enable next-generation digital thoracic phenotyping for personalized cardiovascular and respiratory characterization, risk stratification, and precision medicine.

## 1. Introduction

Thoracic morphology plays a crucial role in determining the spatial relationship between the chest wall, intrathoracic organs, and cardiovascular structures. Variations in chest conformation may influence cardiac position, ventricular filling, myocardial mechanics, respiratory function, and the interpretation of several diagnostic imaging modalities [1].

Among thoracic deformities, pectus excavatum (PE) represents the most common congenital abnormality of the anterior chest wall and is characterized by posterior displacement of the sternum and reduction in the anteroposterior (A–P) thoracic diameter [2,3]. The severity of this deformity is traditionally quantified using the Haller Index (HI), defined as the ratio between the transverse thoracic diameter and the minimum sternum-to-spine distance, typically measured by computed tomography (CT) [4].

Although CT-derived HI remains the reference standard for anatomical assessment of chest wall deformities, its routine use is limited by several practical and clinical considerations. The requirement for ionizing radiation exposure, specialized imaging equipment, dedicated software, and trained personnel reduces its applicability in large-scale screening programs, serial follow-up examinations, outpatient evaluations, and bedside clinical practice [5,6,7]. Similar limitations apply, although to a lesser extent, to chest radiography-based measurements. Consequently, there is increasing interest in developing non-radiological, inexpensive, and easily reproducible methods for assessing thoracic conformation [8,9,10,11,12,13,14].

To address this unmet clinical need, our research group developed and subsequently validated the Modified Haller Index (MHI), a non-invasive anthropometric alternative to the conventional HI [15]. The MHI is calculated as the ratio between the maximum external latero-lateral (L–L) thoracic diameter and the internal A–P thoracic diameter. The maximum external L–L thoracic diameter is measured at nipple level using a dedicated graduated anthropometric caliper, whereas the internal A–P thoracic diameter is obtained by conventional transthoracic echocardiography from the parasternal long-axis view as the distance between the ultrasound entry point at the skin surface and the posterior wall of the descending thoracic aorta, which serves as a surrogate marker of the anterior surface of the vertebral body. This combined anthropometric-echocardiographic approach provides a simple, radiation-free surrogate of the conventional radiological HI while allowing bedside assessment without CT or chest radiography. The original validation study demonstrated a close agreement between MHI and conventional HI measurements, establishing MHI as a reliable radiation-free surrogate marker of thoracic conformation. Since its introduction, the MHI has been progressively applied across a wide range of clinical settings, including PE [16], coronary artery disease (CAD) [17,18,19], mitral valve prolapse (MVP) [20,21], mitral annular disjunction (MAD) [22], obesity [23], pregnancy [24], pulmonary diseases [25], neonatal medicine [26], and atrial fibrillation (AF) [27]. Beyond its role as a thoracic morphometric marker, MHI has also been employed to assess the test–retest reproducibility and intra-observer/inter-observer variability of key echocardiographic parameters, particularly left ventricular ejection fraction (LVEF) and left ventricular global longitudinal strain (LV-GLS), highlighting the influence of chest wall conformation on cardiovascular imaging measurements [28].

The growing body of evidence generated over recent years has demonstrated that thoracic conformation is not merely an anatomical characteristic but an important determinant of cardiopulmonary phenotype. Variations in chest wall geometry may influence cardiac chamber dimensions, ventricular filling, myocardial deformation parameters, ventricular–arterial coupling (VAC), respiratory mechanics, symptom perception, and cardiovascular risk stratification. These observations have substantially expanded the clinical relevance of thoracic morphometry beyond the traditional assessment of chest wall deformities.

Despite its proven feasibility and increasing clinical adoption, MHI assessment still relies on separate measurement tools and operator-dependent procedures, potentially affecting standardization, reproducibility, and large-scale implementation. From a biomedical engineering perspective, the development of dedicated measurement technologies may represent a crucial step toward improving procedural consistency, portability, and clinical usability. In this context, we developed a novel, patented anatomical measurement device specifically designed for standardized thoracic morphometric assessment and determination of the MHI.

In this narrative review, we summarize the historical evolution of thoracic morphometry, the development and validation of the MHI, the engineering rationale and design characteristics of the patented measurement device, and the current evidence supporting the clinical relevance of thoracic conformation across different cardiovascular and respiratory conditions. We also discuss future bioengineering perspectives, including digital integration, artificial intelligence applications, and the potential role of dedicated thoracic morphometric technologies in precision phenotyping and personalized medicine.

## 2. Historical Evolution of Thoracic Morphometry

### 2.1. Conventional Haller Index: Origins and Clinical Applications

The quantitative assessment of thoracic morphology has traditionally relied on radiological imaging techniques. Among the various methods proposed over the last decades, the HI has become the most widely accepted parameter for evaluating the severity of anterior chest wall deformities, particularly PE [29,30,31]. First introduced by Haller and colleagues in 1987 [4], the index is calculated as the ratio between the maximum internal transverse thoracic diameter and the minimum distance between the posterior surface of the sternum and the anterior aspect of the vertebral body.

**Figure 1:**
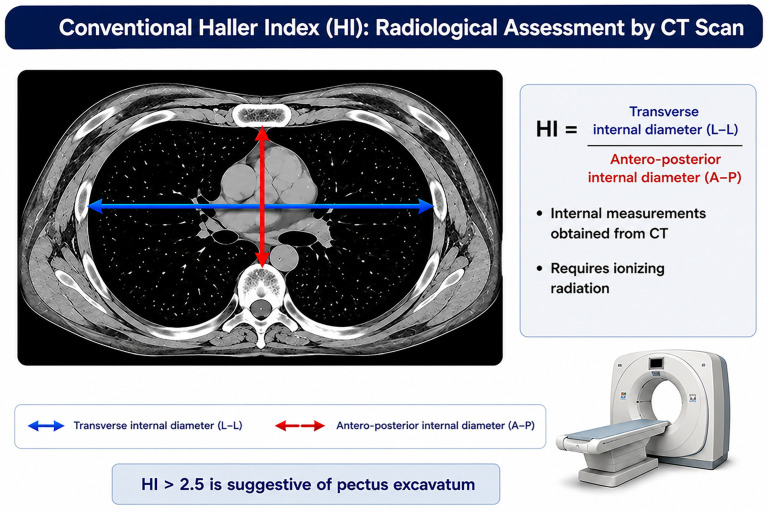
Conventional Haller Index (HI): Radiological Assessment by Computed Tomography. Axial computed tomography image illustrating measurement of the maximum internal transverse thoracic diameter (L–L, blue arrow) and the minimum internal antero-posterior (A–P) distance between the posterior surface of the sternum and the anterior aspect of the vertebral body (red arrow). The conventional Haller Index is calculated as the ratio between the transverse internal thoracic diameter and the internal antero-posterior diameter (HI = L–L/A–P). Because both dimensions are obtained from computed tomography, determination of the HI requires radiological imaging and exposure to ionizing radiation.

As illustrated in Figure 1, both measurements are obtained from axial CT images, allowing quantitative characterization of thoracic shape through calculation of the HI. The figure also highlights the anatomical landmarks used for index determination and the geometric relationship between the transverse and A–P thoracic dimensions.

Since its introduction, the HI has become the reference standard for anatomical characterization of PE and for surgical decision-making. The widespread adoption of CT enabled accurate quantification of chest wall deformity severity and facilitated the establishment of diagnostic and therapeutic thresholds. In clinical practice, an HI value greater than 3.25 has traditionally been considered indicative of severe PE and may support surgical correction in appropriately selected patients [32,33,34].

Beyond its original application, the HI has progressively been used to investigate the relationship between thoracic conformation and cardiopulmonary function. Several studies demonstrated that severe chest wall deformities may alter cardiac position, reduce right ventricular filling, impair exercise performance, and affect respiratory mechanics [35,36,37,38,39,40,41,42,43,44,45,46]. Consequently, thoracic morphometry has evolved from a purely anatomical descriptor to a clinically relevant marker of cardiopulmonary interaction.

### 2.2. Limitations of Radiological Thoracic Morphometry

Despite its recognized clinical value, conventional HI assessment presents several limitations. CT, which remains the reference standard for HI determination, requires exposure to ionizing radiation and is associated with relatively high costs, limited portability, and reduced accessibility outside specialized centers [47,48]. These limitations become particularly relevant when repeated measurements are required, such as during longitudinal follow-up studies, pediatric evaluations, screening programs, or serial assessment of disease progression [49,50,51].

Chest radiography has been proposed as a lower-cost alternative for estimating thoracic morphology; however, it still involves radiation exposure and may be affected by projection-dependent variability, differences in patient positioning, and lower anatomical resolution compared with CT [52,53]. Furthermore, both CT- and radiography-based methods require dedicated imaging facilities and cannot be easily integrated into routine bedside evaluations [54,55].

Recognizing these limitations, several non-radiological methodologies have been developed to quantify chest wall deformities while avoiding exposure to ionizing radiation, particularly in pediatric and young adult populations. Early approaches relied on direct anthropometric measurements of the anterior chest wall using rigid rulers coupled with leveling systems, allowing external assessment of sternal depression [8,9]. Subsequently, more advanced technologies were introduced, including three-dimensional laser scanning [10] and white-light surface scanning systems [11] capable of generating detailed reconstructions of thoracic surface geometry. Another non-radiological approach was proposed by Lain et al. [12], who developed an external three-dimensional scanning platform capable of reconstructing thoracic morphology and deriving external morphometric indices, including an External HI that showed good correlation with conventional CT-derived measurements. More recently, Trò et al. [13] developed a semi-automated image-processing pipeline capable of calculating conventional severity indices while introducing the Volumetric Correction Index (VCI), a novel parameter designed to quantify the volumetric extent of chest wall deformity and potentially improve preoperative assessment. Similarly, Tabard-Fougère et al. [14] demonstrated that the External Haller Index (EHI), obtained through a combination of clinical examination, three-dimensional surface scanning, and magnetic resonance imaging, provides a reliable and accurate estimation of thoracic deformity severity, showing strong agreement with conventional radiological measurements. These techniques demonstrated good agreement with conventional radiological measurements and proved particularly useful for monitoring changes in chest wall morphology before and after corrective surgery for PE, as well as during non-operative treatment and longitudinal follow-up.

Although these non-radiological methods represented important advances, their widespread implementation remained limited by the need for dedicated equipment, specialized software, operator training, or complex image-processing procedures. Even the most recent approaches based on three-dimensional reconstruction, semi-automated image analysis, or external surface scanning still require dedicated hardware, advanced computational processing, and specialized expertise, potentially limiting their applicability in routine bedside assessment and large-scale screening programs. Consequently, many of these techniques remained confined to specialized centers and were not easily applicable in routine clinical practice.

### 2.3. Development and Validation of the Modified Haller Index

The need for a non-radiological approach to thoracic morphometry led our research group to develop the MHI, an anthropometric parameter specifically designed to estimate thoracic conformation without exposure to ionizing radiation. The MHI is calculated as the ratio between the maximum external L–L thoracic diameter and the internal A–P thoracic diameter. The L–L diameter is measured at nipple level as the maximum transverse distance between the two most lateral points of the thoracic cage with the upper limbs abducted approximately 30–45°. The A–P diameter is obtained echocardiographically from the parasternal long-axis view and corresponds to the distance between the ultrasound entry point at the skin surface, which typically overlies the most depressed point of the sternum, and the posterior wall of the descending thoracic aorta visualized behind the left atrium. The posterior wall of the descending aorta serves as a surrogate marker of the anterior surface of the vertebral body, thereby allowing indirect estimation of the sterno-vertebral distance without radiological imaging. The descending thoracic aorta was selected because it is consistently visualized in the parasternal long-axis view and provides a reliable echocardiographic landmark closely related to the anterior surface of the vertebral column.

This methodology was originally developed and validated against the conventional radiological HI, demonstrating excellent feasibility and strong agreement with standard radiological measurements.

**Figure 2:**
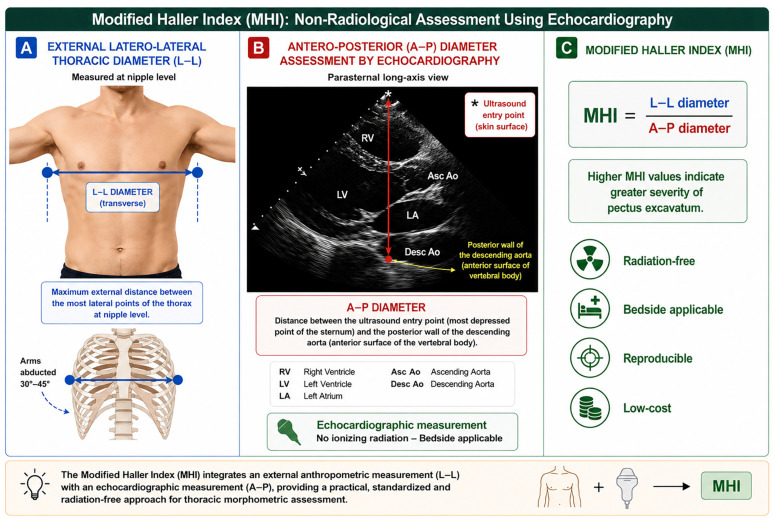
Modified Haller Index (MHI): Non-Radiological Assessment Using Echocardiography. (**A**) Measurement of the maximum external latero-lateral (L–L) thoracic diameter at nipple level using external anthropometric landmarks corresponding to the most lateral points of the thoracic cage. (**B**) Echocardiographic determination of the internal antero-posterior (A–P) thoracic diameter from the parasternal long-axis view. The A–P diameter is measured as the distance between the ultrasound entry point at the skin surface (corresponding to the most depressed point of the sternum) and the posterior wall of the descending aorta, which serves as a surrogate marker of the anterior surface of the vertebral body. (**C**) Calculation of the Modified Haller Index as the ratio between the external latero-lateral thoracic diameter and the internal antero-posterior thoracic diameter (MHI = L–L/A–P). By integrating anthropometric and echocardiographic measurements, the MHI provides a practical, reproducible, bedside-applicable, low-cost, and radiation-free alternative to the conventional radiological Haller Index for thoracic morphometric assessment.

As depicted in Figure 2, MHI determination combines an external anthropometric measurement of the maximum L–L thoracic diameter with an echocardiographic assessment of the internal A–P thoracic diameter obtained from the parasternal long-axis view. This integrated approach enables quantitative characterization of thoracic morphology using readily identifiable anatomical landmarks while avoiding exposure to ionizing radiation.

Unlike the conventional radiological HI, MHI determination can be performed directly during clinical examination using simple, inexpensive, and widely available instruments, allowing immediate bedside assessment and facilitating repeated measurements during longitudinal follow-up.

Following its introduction, the MHI underwent clinical validation against conventional radiological HI measurements. In the original validation study [15], conducted in 100 consecutive subjects undergoing chest radiography, the modified anthropometric index showed excellent agreement with the conventional HI, with no significant difference between mean values (1.99 ± 0.30 vs. 1.93 ± 0.35, *p* = 0.12) and a strong correlation between the two methods (r = 0.81, *p* < 0.0001). Bland–Altman analysis demonstrated narrow limits of agreement (+0.37 to −0.51), supporting the reliability of the non-radiological approach as a surrogate measure of thoracic morphology.

The introduction of the MHI represented a conceptual shift in thoracic morphometry. Rather than being confined to the radiological assessment of congenital chest wall deformities, thoracic shape evaluation became accessible to cardiologists, pulmonologists, internists, sports medicine physicians, obstetricians, and other healthcare professionals involved in the assessment of cardiopulmonary physiology. Subsequent investigations expanded the validation process to heterogeneous clinical populations, including individuals with obesity, PE, pregnancy-related anatomical changes, and various cardiovascular conditions, consistently confirming the feasibility, reproducibility, and clinical applicability of MHI assessment.

A fundamental advantage of the MHI is that it provides information analogous to the conventional radiological HI while avoiding ionizing radiation exposure and reducing dependence on advanced imaging facilities. The principal methodological differences between the two approaches are summarized in Table 1.

## 3. Engineering Development of a Novel Anatomical Measurement Device

### 3.1. Rationale for Device Development

The progressive expansion of thoracic morphometry from the assessment of PE to broader cardiovascular and respiratory applications has generated the need for measurement systems specifically designed for clinical use. Although the MHI has demonstrated good agreement with conventional radiological measurements and has been successfully applied in multiple clinical settings, its determination still relies on the sequential acquisition of two distinct measurements obtained through different procedures. Specifically, the L–L thoracic diameter is measured externally using dedicated anthropometric tools, whereas the A–P thoracic diameter requires echocardiographic visualization of the thoracic structures. The final MHI value is subsequently derived from the ratio between these two measurements.

In routine practice, this approach requires multiple procedural steps, the use of separate instruments, and the integration of measurements obtained from different anatomical perspectives. Such a methodology may introduce variability related to patient positioning, anatomical landmark identification, echocardiographic image acquisition, operator experience, and measurement reproducibility. These limitations may become particularly relevant during serial follow-up examinations, large-scale screening programs, and multicenter clinical studies.

From a bioengineering perspective, the availability of a dedicated device specifically conceived for thoracic morphometric assessment could substantially improve procedural standardization, measurement repeatability, and ease of use. A major conceptual advantage of such a system is the possibility of obtaining, through a single positioning of the device on the thoracic surface, the two geometrical parameters required for direct calculation of a thoracic morphometric index analogous to the conventional HI and MHI, thereby avoiding the separate acquisition and subsequent integration of measurements required by current methodologies.

Furthermore, the development of a portable, inexpensive, and radiation-free system may facilitate the integration of chest shape assessment into routine cardiovascular and respiratory evaluations. The novel patented anatomical measurement device was therefore conceived to provide a dedicated technological solution capable of simplifying thoracic morphometric assessment while maintaining anatomical accuracy, reproducibility, and clinical applicability.

### 3.2. Design Requirements

The design process was guided by a series of clinical and engineering requirements intended to optimize the applicability of thoracic morphometry in routine practice.

First, the device had to permit rapid assessment of thoracic dimensions during routine bedside examination without requiring complex instrumentation or external imaging systems. Second, the measurement procedure had to be reproducible and based on standardized anatomical landmarks. Third, the device had to be portable, lightweight, and suitable for use in different clinical environments, including outpatient clinics, echocardiography laboratories, pulmonary function laboratories, hospital wards, and screening settings.

Additional design requirements included ease of cleaning and maintenance, mechanical robustness, ergonomic handling, and compatibility with repeated measurements performed by different operators. Particular attention was also devoted to minimizing measurement errors related to patient posture, anatomical landmark identification, and operator-dependent positioning.

These design objectives were defined following careful analysis of previously available anatomical measurement systems and their inherent limitations for quantitative thoracic morphometry. The resulting specifications provided the conceptual and technical framework for the development of a dedicated device capable of standardized bedside assessment of thoracic geometry and direct determination of the parameters required for MHI calculation.

### 3.3. Prior Anatomical Measurement Systems and Their Limitations

**Figure 3:**
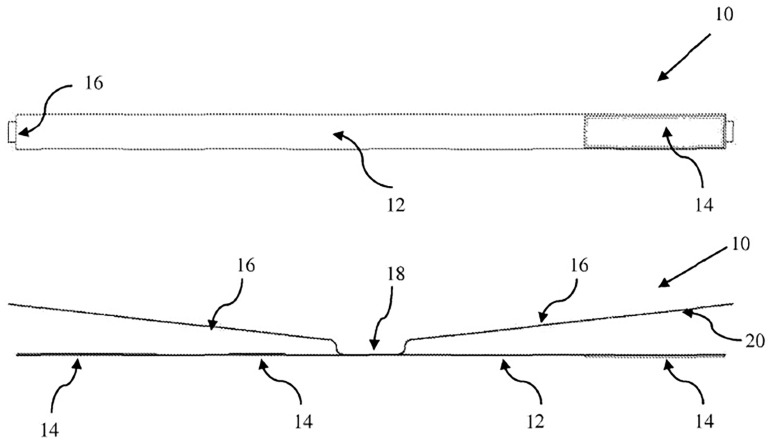
Flexible thoracic measurement assembly (10) previously proposed for external anatomical evaluation. The apparatus comprises a circumferential strap (12) designed to surround the subject’s thorax and secured by a fastening section (14). A graduated measuring tape (16) is attached to the strap at a central attachment position (18) and extends laterally on both sides. The measuring tape incorporates a graduated scale (20), allowing direct determination of distances between the central attachment point and the lateral thoracic boundaries. This configuration was originally developed for assessment of chest rotation and spinal position rather than for quantitative thoracic morphometry or Modified Haller Index (MHI) determination. Adapted from previously described prior-art devices.

**Figure 4:**
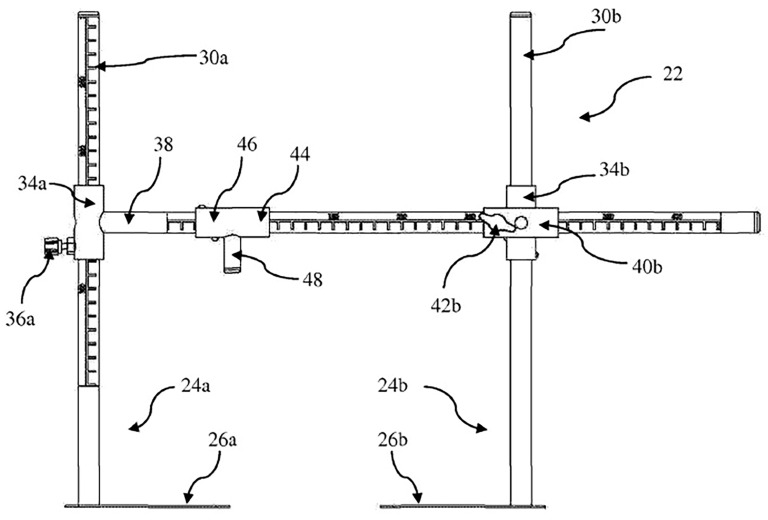
Frontal view of a previously described measurement structure (22) designed for evaluation of chest asymmetry and spinal alignment. The apparatus includes a first support member (24a) and a second support member (24b) mounted on respective base plates (26a,26b). Each support member incorporates a vertical pillar (30a,30b) carrying measurement graduations. The support members are connected by a transverse cross-bar (38) through adjustable connectors (34a,34b) secured by locking screws (36a,42b). A movable pointer assembly (44) incorporating a sleeve (46) and a downward-pointing indicator (48) can slide along the cross-bar to identify anatomical reference points. The movable support member further incorporates a sliding carriage (40b) enabling adjustment of the measurement geometry according to body size. Adapted from previously patented anatomical measurement systems.

**Figure 5:**
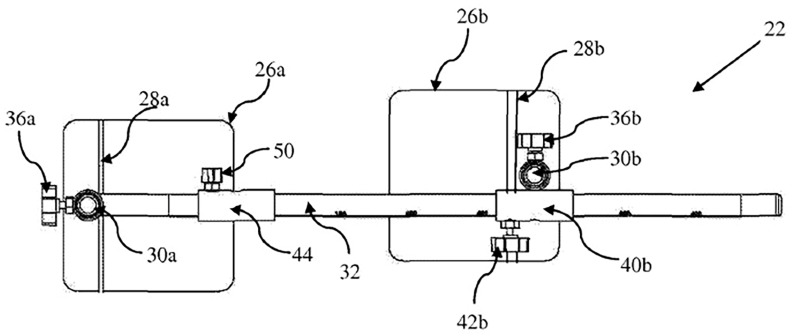
Top view of the measurement structure (22) illustrating the spatial relationship between the principal components. The system comprises two lateral support plates (26a,26b) carrying visual reference markers (28a,28b) and vertical measurement pillars (30a,30b). The pillars are connected by a transverse cross-bar (32) supporting a movable pointer carriage (44) equipped with a locking mechanism (50). The left-side support includes a locking screw (36a) for securing the vertical position of the left adjustable connector on pillar 30a, while the right-side support incorporates an adjustable connector (40b) and locking screws (36b,42b) allowing vertical adjustment of the connector and positional adjustment of the cross-bar. This configuration enables measurement of transverse body asymmetry relative to a central anatomical landmark. Adapted from prior art.

**Figure 6:**
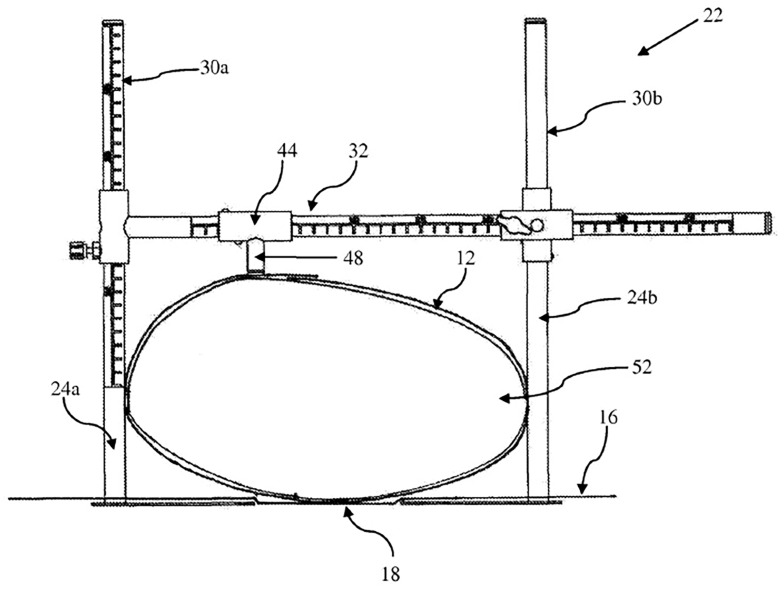
Schematic representation of the measurement procedure using the prior-art apparatus (22). The thorax (52) is positioned between the left and right support members (24a,24b), while the flexible thoracic strap (12) is arranged around the body and aligned with the central attachment point (18). The apparatus is placed on a supporting surface (16), which serves as the reference plane for positioning both the subject and the measurement structure. The vertical pillars (30a,30b) support the transverse measurement bar (32), along which the movable pointer assembly (44) and its downward-pointing indicator (48) are positioned over a selected anatomical landmark. Measurements are obtained relative to external reference structures in order to estimate chest asymmetry and spinal position. Although effective for postural and scoliosis-related assessments, the apparatus was not specifically designed for direct determination of thoracic diameters or calculation of the Modified Haller Index. Adapted from prior art.

The development of dedicated anatomical measurement systems has long represented an important objective in biomedical engineering. Before the introduction of dedicated thoracic morphometric devices, several anatomical measurement systems had been proposed for the assessment of body asymmetry, spinal alignment, and chest wall configuration [56,57,58,59]. Representative examples of these prior-art systems are shown in Figure 3, Figure 4, Figure 5 and Figure 6.

The measurement assembly illustrated in Figure 3 consists of a flexible thoracic strap equipped with a graduated measurement tape and a central attachment point, allowing circumferential assessment of body geometry. This simple configuration was primarily intended to estimate trunk rotation and external body asymmetry through indirect circumferential measurements rather than to provide quantitative assessment of thoracic diameters.

Building upon this concept, a more complex measurement frame was subsequently developed (Figure 4 and Figure 5), incorporating vertical support pillars, adjustable connectors, calibrated scales, movable carriages, and a centrally positioned pointer. These components enabled estimation of thoracic asymmetry and spinal position by measuring geometric relationships between external anatomical landmarks and predefined reference planes.

The operational principle of the prior-art system is illustrated in Figure 6.

During measurement, the subject is positioned within the supporting framework, while the movable pointer is aligned with a selected anterior anatomical landmark. Dimensional measurements are then obtained relative to external reference structures, allowing indirect assessment of body asymmetry and thoracic configuration. The measurement process therefore relies on multiple structural components, sequential positioning steps, and indirect geometric reconstruction of anatomical relationships.

Although these systems represented important advances in external anthropometric assessment, they were primarily conceived for scoliosis monitoring, postural analysis, or spinal alignment evaluation rather than for quantitative thoracic morphometry. Consequently, they require multiple measurement steps, external support structures, and indirect geometric calculations, limiting their applicability for rapid bedside assessment of thoracic dimensions. Furthermore, none of these systems was specifically designed to provide direct determination of the geometrical parameters required for HI or MHI calculation, nor were they developed for routine cardiovascular or respiratory phenotyping.

The patented device described in the present study was specifically designed to overcome these limitations. Unlike previous systems, it directly integrates thoracic anatomical landmarks, measurement scales, movable sliders, and a dedicated sternal reference mechanism into a compact and portable architecture. In contrast to prior-art devices requiring indirect estimation of thoracic geometry, the proposed system enables direct acquisition of the L–L and A–P thoracic diameters within a single standardized mechanical framework. This design enables direct acquisition of the geometrical parameters required for MHI determination while minimizing operator dependency, improving procedural standardization, and facilitating routine clinical implementation.

### 3.4. Structural Architecture of the Device

The novel patented anatomical measurement device described in this review (Italian patent application No. 102025000011096) consists of a planar orthogonal mechanical framework specifically designed for standardized thoracic morphometric assessment.

**Figure 7:**
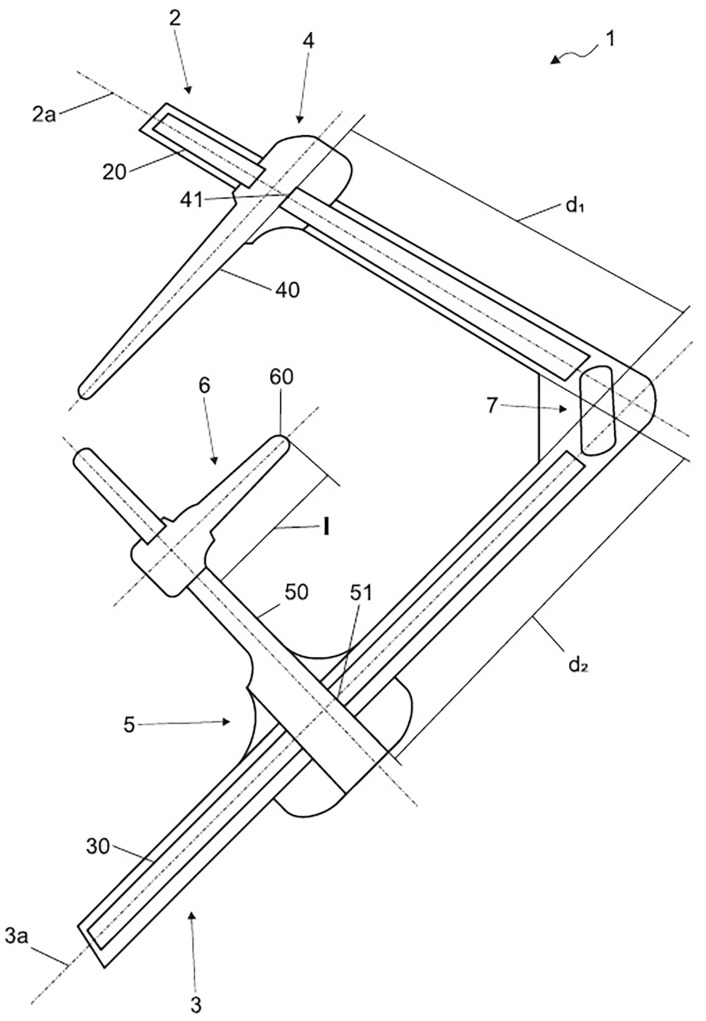
Schematic representation of the patented anatomical measurement device (1) designed for standardized thoracic morphometric assessment. The device comprises a transverse arm (2), positioned posteriorly to the patient and oriented along the first axis (2a), incorporating a graduated scale (20), and a lateral arm (3), oriented along the second axis (3a), orthogonal to the first axis, and incorporating a second graduated scale (30). A first movable slider (4) translates along the transverse arm and carries a first contact edge (40) together with a first reference notch (41), allowing determination of the distance d_1_, defined as the maximum latero-lateral (L–L) thoracic diameter. A second movable slider (5) translates along the lateral arm and carries a second contact edge (50) together with a second reference notch (51), allowing determination of the distance d_2_, corresponding to the measured antero-posterior (A–P) thoracic distance prior to correction for the known length (l) of the sternal pointer. The device further includes a movable sternal pointer (6), ending in a rounded tip (60), which can be positioned directly over the most depressed point of the sternum and has a known length (l). In combination with the posterior reference element and the graduated lateral arm, the known length of the sternal pointer enables direct determination of the anteroposterior (A–P) thoracic diameter. An ergonomic gripping slot (7) facilitates manipulation and stabilization of the device during measurements. The orthogonal arrangement of the transverse and lateral arms forms a rigid square-like framework that enables standardized and reproducible acquisition of the L–L and A–P thoracic diameters required for calculation of the Modified Haller Index (MHI).

As illustrated in Figure 7, the device incorporates two rigid arms arranged along mutually perpendicular axes, forming a stable square-like structure that serves as the reference geometry for measurement acquisition. The transverse arm is positioned posteriorly with respect to the patient and is specifically designed to measure the maximum L–L thoracic diameter through its graduated scale and two movable lateral contact sliders, which are advanced until gentle contact with the most lateral points of the thoracic cage is achieved, thereby providing a direct measurement of the maximum transverse chest dimension. The lateral arm, in contrast, supports a dedicated A–P measurement system integrating both the movable sternal pointer and the posterior vertebral reference element within the same mechanical framework.

Unlike conventional anthropometric calipers, which rely exclusively on external contact surfaces, the sternal pointer introduces a dedicated anatomical referencing mechanism that directly identifies the sternal landmark while maintaining a fixed geometric relationship with the measurement framework. This component represents the core originality of the device, transforming thoracic morphometry from a simple external dimensional assessment into a standardized anatomical measurement procedure.

The combination of the sternal pointer and the posterior reference element creates a dedicated A–P measurement system specifically designed for thoracic morphometry. More specifically, the graduated lateral arm enables direct measurement of the A–P thoracic diameter by taking into account the known length of the sternal pointer. Once the rounded tip of the sternal pointer is positioned on the most depressed point of the sternum and the posterior reference element is advanced until firm contact with the posterior thoracic boundary is achieved, the position of the movable slider along the calibrated lateral arm provides an immediate reading of the A–P thoracic diameter without requiring additional measurements or mathematical corrections. This dual-reference configuration integrates external thoracic landmarks and posterior anatomical references within a single mechanical system, enabling rapid, standardized, and reproducible bedside assessment of thoracic morphology.

**Figure 8:**
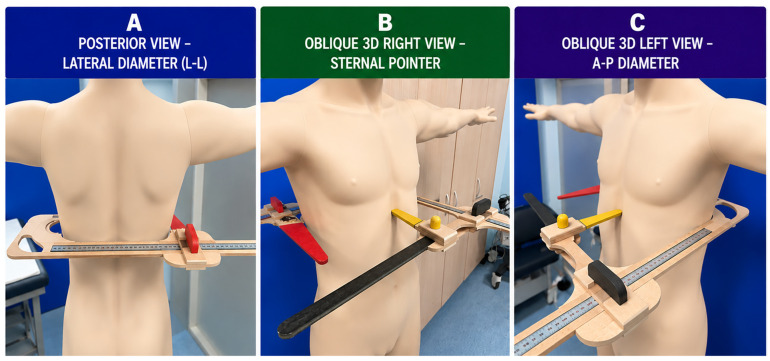
Clinical application of the patented device for bedside measurement of the Modified Haller Index (MHI). (**A**) Posterior view showing measurement of the maximum latero-lateral thoracic diameter (L–L). The lateral arms are positioned at the level of the lower third of the sternum, and the sliding contact elements are advanced until they reach the most lateral points of the thoracic cage. (**B**) Right oblique view illustrating positioning of the patented sternal pointer. The rounded tip of the pointer, mounted on the transverse arm, is advanced perpendicularly to contact the lower third of the sternum, providing a reproducible anterior anatomical landmark. (**C**) Left oblique view demonstrating the complete three-dimensional configuration of the device during measurement of the antero-posterior thoracic diameter (A–P). The lateral arm remains aligned with the mid-axillary line, whereas the sternal pointer projects medially from the transverse arm toward the lower third of the sternum. Simultaneously, the posterior reference element contacts the posterior thoracic surface at the same horizontal level, allowing direct bedside measurement of the A–P thoracic diameter and immediate calculation of the Modified Haller Index (MHI = L–L/A–P).

Figure 8 provides a clinical three-dimensional representation of the patented device during bedside application. The three photographic views illustrate the actual spatial configuration of the instrument and demonstrate how the two thoracic diameters required for MHI calculation are obtained within a single standardized mechanical framework.

The posterior view (Figure 8A) illustrates direct measurement of the maximum L–L thoracic diameter. The posterior transverse arm of the device is positioned behind the patient at the horizontal level corresponding to the lower third of the sternum. The two movable lateral contact elements are advanced until they gently contact the most lateral points of the thoracic cage, allowing direct measurement of the maximum transverse thoracic diameter.

The right oblique view (Figure 8B) highlights the positioning of the patented sternal pointer. The rounded atraumatic tip, mounted on the transverse arm of the device, is advanced medially until it gently contacts the lower third of the sternum, thereby establishing a reproducible anterior anatomical landmark. This view clearly demonstrates that the sternal pointer originates from the transverse arm, whereas the lateral arm remains positioned laterally at the level of the mid-axillary line.

The left oblique view (Figure 8C) illustrates the complete three-dimensional measurement configuration. The lateral arm remains aligned with the lateral aspect of the thoracic cage, while the sternal pointer projects perpendicularly from the transverse arm toward the lower third of the sternum. Simultaneously, the posterior reference element is advanced until it gently contacts the posterior thoracic surface at the same horizontal level as the sternal pointer, thereby defining the A–P thoracic diameter. The graduated lateral arm provides the direct A–P measurement using the fixed and known length (l) of the sternal pointer as the geometric reference, allowing immediate determination of the A–P thoracic diameter without additional measurements, mathematical corrections, or radiological imaging.

By integrating both thoracic dimensions within a single mechanical framework, the device enables immediate bedside calculation of the Modified Haller Index (MHI = L–L/A–P) without the need for separate measurement procedures or subsequent integration of data obtained from different instruments or imaging modalities. Unlike conventional anthropometric calipers or generic body-measurement devices, the present instrument was specifically designed for thoracic morphometry and incorporates dedicated structural components that improve anatomical alignment, measurement standardization, reproducibility, and operator ergonomics. The rigid orthogonal configuration—including the posterior transverse arm, the graduated lateral arm, the patented sternal pointer, and the posterior reference element—minimizes positional variability while enabling rapid, standardized, and reproducible acquisition of both thoracic diameters required for bedside MHI determination.

### 3.5. Measurement Workflow

**Figure 9:**
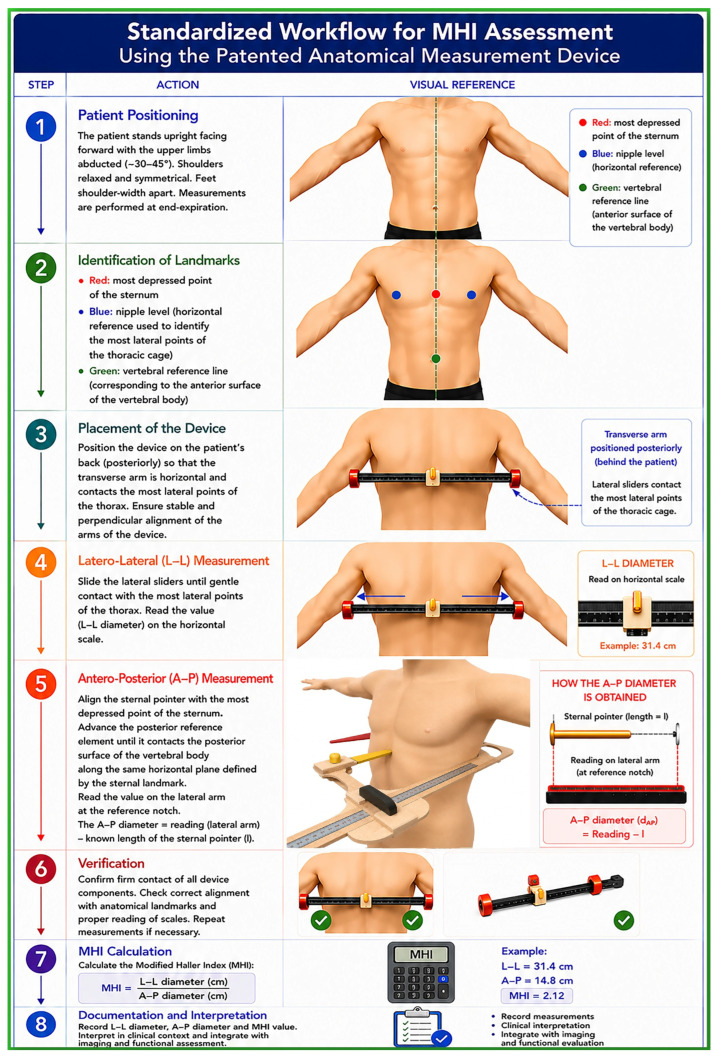
Standardized workflow for non-invasive thoracic morphometric assessment using the patented anatomical measurement device. The procedure consists of eight sequential steps: (1) patient positioning in the standing position with the upper limbs slightly abducted (approximately 30–45°); (2) identification of the anatomical landmarks, including the deepest point of the lower third of the sternum, the horizontal reference level used to identify the most lateral points of the thoracic cage, and the corresponding posterior reference level; (3) positioning of the device behind the patient’s thorax, with the posterior transverse arm placed at the same horizontal level as the sternal landmark and the lateral contact sliders aligned with the most lateral points of the thoracic cage; (4) acquisition of the maximum latero-lateral (L–L) thoracic diameter by advancing the lateral contact sliders until gentle contact with the thoracic cage is achieved and reading the value directly from the graduated posterior transverse arm; (5) acquisition of the antero-posterior (A–P) thoracic diameter by positioning the rounded tip of the sternal pointer on the lower third (deepest point) of the sternum and advancing the posterior reference element until gentle contact with the posterior thoracic surface is achieved at the same horizontal level. The A–P thoracic diameter is then obtained directly from the graduated lateral arm using the known length (l) of the sternal pointer as the geometric reference (A–P diameter = reading − l); (6) verification of correct device positioning, anatomical alignment, and scale readings; (7) calculation of the Modified Haller Index according to the formula MHI = L–L diameter/A–P diameter; and (8) documentation and clinical interpretation of the measurements.

Figure 9 illustrates the complete measurement pathway for MHI determination using the patented anatomical measurement device.

The proposed protocol was specifically developed to ensure reproducible, operator-independent, and anatomically standardized thoracic morphometric assessment across different clinical settings. The workflow comprises eight sequential steps encompassing patient preparation, identification of the relevant anatomical landmarks, device positioning, acquisition of the L–L and A–P thoracic diameters, verification of measurement accuracy, immediate calculation of the MHI, and final documentation of the results.

Standardization of patient positioning, anatomical landmark identification, and device alignment minimizes operator-dependent variability and ensures consistent acquisition of both thoracic diameters. The integrated mechanical design allows the L–L and A–P measurements to be obtained during a single examination using the same reference framework, thereby avoiding the use of separate measuring instruments, mathematical corrections, or radiological imaging. The direct determination of the A–P thoracic diameter through the patented sternal pointer and posterior vertebral reference element further enhances procedural consistency and represents the principal engineering innovation of the proposed system.

Following verification of correct device positioning and scale readings, the MHI is calculated immediately as the ratio between the L–L and A–P thoracic diameters, enabling real-time bedside thoracic morphometric assessment. The standardized workflow facilitates rapid and reproducible data acquisition while reducing measurement variability, making the device suitable for serial follow-up examinations and multicenter clinical studies.

Owing to its portability, simplicity, low manufacturing cost, and radiation-free operation, the proposed system is readily applicable in outpatient clinics, echocardiography and pulmonary function laboratories, hospital wards, and large-scale screening programs. By combining standardized acquisition with immediate index calculation, the device may facilitate the routine incorporation of thoracic morphometry into cardiovascular and respiratory phenotyping and provide a practical platform for future digital integration and automated morphometric analysis.

## 4. Clinical Applications of the Modified Haller Index

Following its introduction as a non-radiological surrogate of thoracic morphology, the MHI has progressively evolved from a simple tool for the assessment of chest wall conformation to a broader instrument for investigating the interaction between thoracic anatomy and cardiopulmonary physiology. Increasing evidence suggests that variations in thoracic geometry may influence cardiac chamber dimensions, ventricular mechanics, exercise hemodynamics, pulmonary function, symptom perception, and the interpretation of cardiovascular imaging findings. Consequently, thoracic morphometry has attracted growing interest as a potential modifier of cardiopulmonary phenotype across a variety of clinical settings.

The following sections summarize the principal clinical conditions in which thoracic morphology and MHI assessment have been investigated, highlighting their potential implications for diagnosis, functional evaluation, risk stratification, and longitudinal follow-up.

### 4.1. Pectus Excavatum and Chest Wall Deformities

PE represents the clinical condition in which thoracic morphometry has been most extensively investigated.

The introduction of the MHI represented an important advance in thoracic morphometry by providing a simple, non-invasive, radiation-free, and bedside-applicable alternative to conventional radiological assessment. By combining external thoracic measurements with echocardiographic determination of the sterno-vertebral distance, MHI enables rapid characterization of thoracic conformation without the need for CT or chest radiography. Validation studies demonstrated a strong agreement between MHI and conventional HI measurements, supporting its use as a reliable surrogate marker of thoracic geometry [15].

Beyond facilitating the assessment of PE severity, the availability of a repeatable and radiation-free morphometric parameter has substantially expanded the clinical role of thoracic conformation analysis. Unlike conventional radiological indices, MHI can be incorporated into routine cardiovascular and respiratory evaluations, allowing serial assessment of thoracic geometry during longitudinal follow-up and across different physiological and pathological conditions.

Importantly, accumulating evidence indicates that PE should not be regarded merely as a localized chest wall abnormality. Progressive reduction in the A–P thoracic diameter may induce external cardiac compression, cardiac displacement and rotation, ventricular preload limitation, altered chamber geometry, and modifications of myocardial mechanics [60,61,62,63,64,65,66]. These adaptations may influence cardiac chamber dimensions, stroke volume, myocardial deformation parameters, exercise performance, respiratory physiology, and symptom perception, even in the absence of intrinsic cardiopulmonary disease [67].

From this perspective, the MHI has progressively evolved from a simple morphometric surrogate of PE severity into a broader marker of cardiopulmonary phenotype. Increasing MHI values identify individuals characterized by a distinctive anatomical and functional profile, including reduced thoracic depth, greater cardiac constraint, smaller ventricular cavities, altered myocardial deformation patterns, and variable degrees of restrictive respiratory physiology. Consequently, thoracic morphometry should be considered an integral component of the multidimensional evaluation of patients with chest wall deformities and a potentially valuable tool for cardiovascular and respiratory phenotyping beyond the traditional assessment of PE severity.

### 4.2. Cardiac Chamber Geometry and Hemodynamic Consequences

The thoracic cage represents the anatomical container within which the heart, great vessels, and lungs interact continuously. Consequently, alterations in chest wall geometry may substantially influence cardiac chamber dimensions, ventricular filling, stroke volume, cardiac output, and overall hemodynamic performance [68,69,70].

Several investigations performed in individuals with PE and other concave-shaped thoracic conformations demonstrated that progressive reduction in the A–P thoracic diameter is associated with smaller right- and left-sided cardiac chamber dimensions despite preserved conventional indices of systolic function [71,72,73,74,75]. These findings suggest that thoracic morphology may directly affect cardiac geometry through external mechanical constraints rather than through intrinsic myocardial disease.

The right ventricle appears particularly vulnerable to thoracic compression because of its anterior position immediately behind the sternum [76]. Mechanical reduction in the retrosternal space may impair right ventricular filling, reduce right ventricular cavity dimensions, and ultimately decrease stroke volume and cardiac output [77,78,79,80]. Consistently, both echocardiographic and cardiac magnetic resonance studies have demonstrated reduced ventricular volumes, diminished stroke volume, and varying degrees of right ventricular functional impairment in subjects with increasing severity of chest wall deformity [81,82,83,84]. Importantly, many of these abnormalities improve after surgical correction of the thoracic deformity, supporting a predominantly mechanical origin [85,86,87,88,89,90].

Left ventricular geometry may also be affected. Reduction in the A–P thoracic diameter may induce leftward displacement, axial rotation, and altered spatial orientation of the heart within the thoracic cavity [91,92,93]. These geometric adaptations can modify ventricular interaction, influence intracardiac flow dynamics, and affect the echocardiographic appearance of both regional and global cardiac function.

Importantly, the MHI provides a quantitative estimate of the degree of thoracic narrowing responsible for these geometric and hemodynamic adaptations. Increasing MHI values identify individuals with progressively narrower thoraces, greater external cardiac compression, smaller cardiac chamber dimensions, and lower stroke volume. Therefore, thoracic morphometry should be considered not merely an anatomical descriptor but also a determinant of cardiovascular structure and hemodynamic phenotype.

### 4.3. Influence of Chest Wall Conformation on Myocardial Strain Imaging

Among the various clinical applications of the MHI, its relationship with myocardial deformation imaging has emerged as one of the most extensively investigated and clinically relevant fields.

Speckle-tracking echocardiography (STE) has become an integral component of contemporary cardiovascular imaging, enabling quantitative assessment of myocardial mechanics through parameters such as LV-GLS, global circumferential strain (GCS), global radial strain (GRS), and right ventricular free-wall longitudinal strain (RV-FWLS) [94,95,96]. These indices are widely recognized as sensitive markers of subclinical myocardial dysfunction and frequently provide incremental information beyond conventional measures of systolic performance [97,98,99,100,101].

However, accumulating evidence indicates that myocardial strain parameters are influenced not only by intrinsic myocardial contractility but also by extracardiac anatomical factors, particularly thoracic geometry. Studies performed in subjects with PE demonstrated that increasing HI values are associated with progressive attenuation of both left- and right-ventricular strain parameters despite preserved LVEF and normal conventional echocardiographic indices [16,44,102,103,104,105,106]. Importantly, the severity of strain impairment was strongly correlated with the degree of thoracic constriction, indicating a direct relationship between chest wall morphology and myocardial deformation measurements.

Subsequent investigations extended these observations to other populations characterized by reduced A–P thoracic diameter, including patients with MVP [22], obese individuals [23], healthy pregnant women [24] and neonates [26]. Across these diverse clinical settings, higher MHI values were consistently associated with lower myocardial strain measurements, suggesting the existence of a common biomechanical mechanism linking thoracic conformation to ventricular mechanics.

Several pathophysiological mechanisms have been proposed to explain the relationship between thoracic morphology and myocardial deformation. Current evidence supports the hypothesis that the reduction in strain magnitude observed in subjects with increased HI is primarily related to extrinsic cardiac compression caused by anterior chest wall constriction rather than to intrinsic myocardial dysfunction. A reduced A–P thoracic diameter may mechanically constrain cardiac expansion, alter ventricular geometry, modify loading conditions, and induce cardiac displacement and rotation within the thoracic cavity. These geometric and mechanical effects appear to predominantly affect basal myocardial segments, where external compression is greatest [62].

Supporting this interpretation, studies performed in individuals with elevated HI consistently demonstrated a concomitant reduction in longitudinal, circumferential, and radial strain parameters, a pattern that differs from the typical evolution of intrinsic myocardial disease, in which impairment of longitudinal deformation generally precedes alterations in circumferential and radial mechanics [107,108,109,110,111,112]. Furthermore, despite the reduction in global strain values, the physiological apex-to-base gradient of myocardial deformation is generally preserved in subjects with thoracic constriction, suggesting maintenance of normal intrinsic myocardial function [113,114,115]. An additional argument supporting a geometry-related mechanism is the strong inverse relationship observed between MHI and strain magnitude across different populations, whereas such associations are absent in subjects with normal thoracic conformation [16,24,26].

Perhaps the most compelling evidence derives from studies demonstrating significant improvement of myocardial strain parameters immediately after surgical correction of pectus deformities. The rapid normalization of deformation indices following restoration of thoracic geometry strongly suggests that the observed abnormalities are largely attributable to reversible mechanical constraints imposed by chest wall morphology rather than to permanent alterations of myocardial contractility [104]. In addition, technical factors related to image acquisition, cardiac orientation, and speckle-tracking analysis may further contribute to the apparent reduction in deformation parameters, particularly in individuals with marked chest wall abnormalities [116,117,118,119].

These observations challenge the traditional assumption that abnormal strain values invariably reflect myocardial disease. In selected populations with altered thoracic morphology, reduced strain measurements may represent, at least in part, the consequence of mechanical and geometric constraints rather than true myocardial dysfunction.

Therefore, chest wall conformation should be regarded as a potential confounding variable in the interpretation of myocardial deformation imaging. Preliminary assessment of thoracic morphology using MHI may facilitate a more accurate interpretation of strain findings and help distinguish geometry-related alterations in myocardial mechanics from genuine myocardial pathology. From this perspective, MHI may serve not only as a marker of chest wall anatomy but also as an important modifier of cardiovascular imaging results and their clinical interpretation.

### 4.4. Exercise Stress Echocardiography and Coronary Artery Disease

Among the various cardiovascular applications of thoracic morphometry, exercise stress echocardiography (ESE) represents one of the clinical settings in which chest wall geometry appears to have particularly relevant diagnostic implications.

Although ESE is widely recognized as an accurate non-invasive technique for the detection of obstructive CAD [120,121,122], false-positive findings remain a well-documented limitation. Indeed, a proportion of patients exhibiting exercise-induced regional wall motion abnormalities subsequently show no evidence of significant epicardial coronary stenosis at invasive coronary angiography. Several clinical factors have been associated with an increased likelihood of false-positive ESE results, including female sex, low pre-test probability of CAD, hypertensive response to exercise, pre-existing resting wall motion abnormalities, and the occurrence of exercise-induced Takotsubo-like contractile patterns [123,124,125,126,127,128,129,130,131,132,133,134,135,136,137,138].

Our group was among the first to demonstrate that specific thoracic morphometric characteristics, namely a reduced A–P thoracic diameter (≤13.5 cm), increased MHI values (>2.5), and a concave chest wall configuration, may predispose individuals to the development of stress-induced regional wall motion abnormalities despite the absence of angiographically significant CAD [17,18].

The proposed mechanism is predominantly mechanical rather than ischemic. A reduced A–P thoracic diameter may increase external cardiac compression and alter ventricular geometry, resulting in paradoxical septal motion, dynamic ventricular dyssynchrony, and abnormal myocardial excursion during physical exercise. These phenomena may become more pronounced during stress conditions and can be misinterpreted as inducible myocardial ischemia. Such findings appear particularly common in women, in individuals with small ventricular cavities, in MVP individuals and in those characterized by a low pre-test probability of CAD [19].

A systematic review evaluating the determinants of false-positive ESE results identified elevated MHI values (>2.5) among the recognized causes of false-positive stress echocardiographic findings [139]. In this context, thoracic morphometry may provide valuable information for interpreting stress-induced wall motion abnormalities and refining the estimation of CAD probability.

Importantly, the prognostic implications appear to differ substantially from the diagnostic findings. In symptomatic patients with MVP and elevated MHI values, positive ESE results were frequently not associated with obstructive CAD at subsequent coronary angiography [19]. Moreover, individuals with narrow A–P thoracic diameters (≤13.5 cm) and elevated MHI values (>2.5) generally exhibited a favorable medium-term clinical outcome, suggesting that chest wall compression may generate apparent functional abnormalities in the absence of clinically relevant myocardial ischemia [17,18].

These observations support the incorporation of thoracic morphometric assessment into the interpretation of stress echocardiography, particularly in patients with low coronary risk profiles, where chest wall geometry may represent an important determinant of exercise-induced imaging abnormalities.

### 4.5. Mitral Valve Prolapse and Mitral Annular Disjunction

MVP is among the cardiovascular disorders in which the influence of thoracic conformation has been most consistently recognized [140]. Beginning with the earliest investigations conducted during the 1970s, numerous clinical and imaging studies reported a frequent coexistence of MVP with thoracic and skeletal abnormalities, including PE, pectus carinatum, scoliosis, straight back syndrome, and connective tissue disorders such as Marfan syndrome [141,142,143,144,145,146,147,148,149,150,151,152,153,154,155,156,157,158,159,160,161]. These phenotypic features are often associated with a reduced A–P thoracic diameter, a flattened or concave chest configuration, and varying degrees of pectus-like deformity.

Over the past decade, increasing attention has been directed toward the assessment of myocardial deformation in MVP. Several studies have shown that the impairment of longitudinal myocardial mechanics observed in MVP patients is predominantly regional rather than global, with preferential involvement of the basal inferolateral left ventricular segments and relative preservation of apical deformation, resulting in a characteristic apical-sparing pattern [162,163,164,165,166,167,168,169,170,171,172,173,174].

While these investigations primarily focused on the distribution and potential arrhythmogenic significance of myocardial deformation abnormalities, our group was among the first to explore the potential contribution of thoracic morphology to this phenomenon. Specifically, we demonstrated that elevated MHI values and reduced A–P thoracic diameters are associated with lower LV-GLS, impaired GCS, and abnormal left ventricular twist mechanics despite preserved conventional systolic function [20]. Notably, these abnormalities predominantly involved the basal and mid-ventricular segments, whereas apical deformation remained relatively preserved, a distribution pattern compatible with the preferential mechanical effects of anterior chest wall compression. The magnitude of strain impairment was found to correlate inversely with thoracic depth and directly with the severity of chest wall constriction, supporting the hypothesis that altered thoracic geometry may generate apparent myocardial dysfunction through cardiac compression, displacement, and geometric remodeling rather than intrinsic myocardial disease.

More recently, attention has focused on the relationship between thoracic conformation and MAD, a structural abnormality increasingly recognized as a potential arrhythmogenic substrate [175,176,177,178,179,180,181,182]. Preliminary observations demonstrated that MVP patients with MAD frequently exhibit a narrow A–P thoracic diameter and elevated MHI values [22]. In this setting, the reduction in myocardial strain parameters may largely reflect chest wall compression rather than myocardial fibrosis or irreversible structural damage.

Importantly, chest wall morphology may also have prognostic implications. In symptomatic MVP patients with moderate mitral regurgitation, elevated MHI values and reduced A–P thoracic diameters were associated with a lower prevalence of significant exercise-induced abnormalities, a low probability of obstructive CAD, and a favorable medium-term clinical outcome [21,183,184,185,186,187,188]. Furthermore, MVP patients with elevated MHI values and reduced A–P thoracic diameters frequently exhibit mild-to-moderate, late-systolic mitral regurgitation that is often not hemodynamically significant despite the presence of symptoms. In these individuals, the severity of mitral regurgitation may occasionally be overestimated because of the combination of small left ventricular and left atrial chamber dimensions, altered cardiac orientation within the thoracic cavity, and geometric constraints imposed by the narrow thorax. Consequently, the hemodynamic burden of mitral regurgitation is generally limited, and exercise-induced progression to severe regurgitation appears relatively uncommon [188]. In these individuals, symptoms such as atypical chest pain, dyspnea, palpitations, exercise-induced electrocardiographic abnormalities, and apparent reductions in myocardial strain may therefore reflect the mechanical consequences of thoracic conformation rather than the presence of advanced valvular or myocardial disease. This concept is supported by the observation that patients with elevated MHI values generally exhibit smaller cardiac chamber dimensions, preserved biventricular systolic function, and a remarkably low incidence of major adverse cardiovascular events during follow-up.

Recent findings also suggest that individuals presenting with MVP and/or MAD in the presence of a narrow A–P thoracic diameter may represent a distinct low-risk phenotype characterized by apparent mechanical impairment of myocardial deformation, limited arrhythmic burden, absence of replacement myocardial fibrosis on cardiac magnetic resonance imaging, and a relatively favorable prognosis [189]. From a pathophysiological standpoint, chest wall constriction may act as an important modifier of disease expression, generating symptoms and imaging abnormalities that mimic a more severe cardiovascular phenotype despite the absence of significant myocardial dysfunction, myocardial fibrosis, or adverse cardiac remodeling. In these patients, the attenuation of myocardial strain parameters may therefore predominantly reflect extrinsic mechanical compression and altered cardiac geometry rather than an underlying arrhythmogenic cardiomyopathic process. Consequently, preliminary assessment of thoracic morphology may contribute to a more accurate interpretation of symptoms, electrocardiographic findings, ESE results, cardiac magnetic resonance findings, and myocardial strain measurements in MVP patients.

This hypothesis may also help explain the apparent paradox frequently observed in clinical practice, whereby MVP subjects with narrow chest configuration often report symptoms disproportionate to the severity of mitral regurgitation while simultaneously exhibiting an overall benign clinical course. In these patients, thoracic geometry itself may represent an important determinant of symptom generation and cardiovascular phenotype expression.

This emerging concept further supports the role of thoracic morphometry as a clinically relevant modifier of cardiovascular phenotype expression beyond its traditional application in chest wall deformities.

### 4.6. Pregnancy

Pregnancy is characterized by profound anatomical and physiological adaptations involving both the cardiovascular and respiratory systems. Progressive elevation of the diaphragm, enlargement of the thoracic cage, and modifications in cardiac position may influence cardiac geometry, ventricular filling, stroke volume, and echocardiographic measurements [190,191,192].

Growing evidence indicates that pregnancy itself may affect myocardial deformation parameters, even in otherwise healthy women. Several studies have reported a progressive reduction in myocardial strain indices throughout gestation, particularly during the third trimester, despite preserved conventional measures of systolic function [193,194,195,196,197]. These observations have been largely attributed to the complex interplay between altered loading conditions, increased circulating blood volume, ventricular remodeling, and the substantial anatomical changes occurring during pregnancy.

Expanding upon these observations, our group demonstrated that thoracic conformation may represent an additional determinant of myocardial mechanics during pregnancy. Specifically, women with elevated MHI values (>2.5), reflecting a reduced A–P thoracic diameter, exhibited lower myocardial deformation parameters despite preserved LVEF [24]. These findings support the hypothesis that altered chest wall geometry and extrinsic cardiac compression may influence myocardial mechanics independently of intrinsic myocardial dysfunction. Furthermore, thoracic conformation appears to affect VAC parameters. Pregnant women with higher MHI values demonstrate a significantly lower stroke volume index and a less pronounced physiological increase in stroke volume throughout gestation, despite similar blood pressure values, arterial elastance, and indices of myocardial contractility. Consequently, alterations in VAC observed in these individuals may reflect mechanical constraints imposed by a reduced A–P thoracic diameter rather than true cardiovascular impairment.

Because radiation-based techniques are generally avoided during pregnancy, MHI assessment offers a particularly attractive non-invasive approach for evaluating thoracic morphology. Incorporating thoracic morphometry into cardiovascular assessment may improve the interpretation of both myocardial strain and hemodynamic parameters, helping to distinguish genuine cardiac dysfunction from geometry-related mechanical effects during normal pregnancy.

### 4.7. Obesity

The relationship between obesity and cardiovascular mechanics is complex and multifactorial. Increased adiposity may alter thoracic geometry through both chest wall and abdominal mechanisms, potentially affecting cardiac dimensions, respiratory function, and cardiovascular imaging findings. In individuals with obesity, the thorax often assumes a more circular configuration characterized by an increased A–P diameter and altered thoraco-abdominal interactions [198].

Studies evaluating the MHI in obese subjects demonstrated that myocardial deformation abnormalities may occur despite preserved LVEF and the absence of overt structural heart disease. In a prospective cohort of individuals with obesity, LV-GLS was impaired in the majority of participants, whereas conventional systolic indices remained within normal limits [23]. Importantly, waist circumference and A–P thoracic diameter emerged as independent predictors of reduced myocardial strain, while traditional cardiovascular risk factors, biochemical variables, and left ventricular mass did not retain independent associations.

These findings support the hypothesis that extrinsic thoraco-abdominal compression may contribute substantially to the apparent impairment of myocardial deformation parameters observed in obesity. Of particular interest, the reduction in strain values appears to be most pronounced in individuals with android obesity, in whom increased abdominal circumference, visceral adiposity, epicardial fat accumulation, and altered thoracic geometry may act synergistically to modify cardiac mechanics. Conversely, the relative preservation of myocardial deformation observed in gynoid obesity suggests that lower-body fat accumulation exerts considerably less mechanical influence on cardiac function [199,200,201].

Collectively, these findings suggest that impaired myocardial deformation should not be considered an inevitable consequence of obesity. The relative preservation of cardiac mechanics observed in individuals with gynoid obesity is consistent with the concept of metabolically healthy obesity (MHO), indicating that excess adiposity per se may not necessarily result in intrinsic myocardial dysfunction when visceral fat accumulation, adverse metabolic profiles, and unfavorable thoraco-abdominal mechanical interactions are absent or limited [202,203,204]. Accordingly, part of the strain abnormalities reported in obese populations may reflect the combined effects of central adiposity, thoraco-abdominal compression, and altered cardiac geometry rather than a true obesity-related cardiomyopathy.

### 4.8. Pulmonary Diseases

Thoracic morphology influences not only cardiovascular mechanics but also respiratory physiology. The interaction between chest wall geometry, lung expansion, diaphragmatic motion, and respiratory muscle function may substantially affect pulmonary performance and contribute to functional limitation.

Evidence from patients with idiopathic pulmonary fibrosis (IPF) indicates that an elevated MHI, reflecting a reduced A–P thoracic diameter and a pectus-like chest configuration, is associated with a more severe restrictive ventilatory pattern [25]. Individuals with higher MHI values exhibit significantly lower forced vital capacity (FVC), total lung capacity (TLC), and diffusing capacity for carbon monoxide (DLCO), together with reduced exercise tolerance and greater oxygen desaturation during exertion. Notably, MHI demonstrates a strong inverse relationship with key spirometric parameters, suggesting that thoracic narrowing may contribute directly to respiratory impairment.

The proposed mechanism involves chronic mechanical constraint of lung expansion and increased stress on the pulmonary parenchyma, which may exacerbate the restrictive physiology already imposed by the underlying pulmonary disease [205]. These findings support the concept that thoracic conformation represents an important extrinsic determinant of respiratory function, acting in parallel with intrinsic pulmonary pathology.

Beyond functional assessment, thoracic morphology also appears to carry prognostic significance. In patients with IPF, elevated MHI values have been independently associated with a higher risk of hospitalization and mortality, identifying a subgroup with a less favorable clinical course [25]. Therefore, MHI may help recognize a distinct cardiopulmonary phenotype characterized by greater physiological impairment and worse outcomes.

The integration of thoracic morphometry into respiratory evaluation may provide additional information regarding disease severity, functional limitation, risk stratification, and longitudinal follow-up, particularly in disorders characterized by restrictive ventilatory physiology.

### 4.9. Neonatology and Early-Life Cardiovascular Phenotyping

The influence of thoracic conformation appears to begin very early in life. Investigations conducted in neonates demonstrated that chest wall morphology is closely associated with cardiac geometry and myocardial deformation parameters, even in the absence of congenital heart disease or overt cardiovascular dysfunction [26]. Infants with a higher MHI, reflecting a reduced A–P thoracic diameter and pectus-like chest configuration, exhibited significantly smaller cardiac chamber dimensions together with marked reductions in left and right ventricular strain parameters despite preserved conventional systolic function. Importantly, myocardial deformation abnormalities were strongly correlated with the severity of thoracic narrowing, indicating that chest wall morphology may directly influence cardiac mechanics from birth. These findings suggest that altered myocardial strain in this setting primarily reflects extrinsic cardiac compression and electromechanical dyssynchrony rather than intrinsic myocardial disease.

The persistence of these associations during early postnatal growth further supports a causal relationship between thoracic geometry and cardiac performance. This mechanistic interpretation is further supported by developmental studies demonstrating that, from the perinatal period through early infancy, thoracic growth is characterized by a predominant increase in the L–L thoracic diameter relative to the A–P diameter. As previously reported by other investigators [206,207], this physiological remodeling progressively modifies thoracic shape during early life and may contribute to the observed changes in cardiac dimensions and myocardial deformation parameters. These observations are particularly relevant because they indicate that chest shape is not merely a consequence of aging, disease, or acquired remodeling but may represent a fundamental anatomical determinant of cardiopulmonary physiology throughout life.

The ability of MHI to identify distinct cardiovascular phenotypes from the neonatal period reinforces the concept that thoracic morphometry may contribute to cardiovascular characterization long before the development of clinically overt disease.

The availability of a simple, non-invasive, radiation-free assessment of thoracic morphology may facilitate future investigations into developmental cardiovascular physiology, pediatric chest wall disorders, and the lifelong impact of thoracic conformation on cardiac structure and function.

### 4.10. Atrial Fibrillation and Emerging Cardiovascular Applications

More recently, thoracic morphometry has been investigated in patients with AF and other cardiovascular disorders, revealing clinically relevant associations that extend beyond the assessment of chest wall anatomy. Studies evaluating the MHI in patients with persistent AF demonstrated that thoracic conformation may significantly influence symptom perception, cardiac geometry, and the overall cardiovascular phenotype [27]. In particular, a high MHI, reflecting a narrow A–P thoracic diameter and a more concave chest configuration, was associated with increased perception of palpitations, dyspnea, and arrhythmia-related symptoms despite relatively limited structural cardiac impairment. Conversely, a low MHI, reflecting a wider A–P thoracic diameter and a more circular thoracic configuration, identified patients who were frequently asymptomatic despite exhibiting a higher burden of cardiovascular risk factors, greater comorbidity, larger cardiac chamber dimensions, impaired biventricular function, reduced atrial mechanical performance, and a more advanced stage of cardiovascular disease [27].

These observations suggest that thoracic morphology may modulate the interaction between cardiac mechanics and symptom perception. Consequently, MHI assessment may help distinguish patients with heightened cardiac awareness from those with reduced symptom perception despite more severe underlying cardiovascular abnormalities. This concept is particularly relevant in AF, where asymptomatic presentations are frequently associated with delayed diagnosis and worse clinical outcomes [208,209,210,211].

### 4.11. Measurement Variability and Standardization

The interpretation of cardiovascular imaging findings requires careful consideration of the factors that may influence measurement accuracy and reproducibility. Among these, thoracic morphology has emerged as a potentially important but frequently overlooked determinant of variability in echocardiographic assessment. Because LVEF and LV-GLS play a central role in diagnosis, risk stratification, therapeutic decision-making, and longitudinal follow-up, understanding the sources of variability affecting these parameters is of considerable clinical relevance [212,213].

Previous investigations demonstrated that individuals with a reduced A–P thoracic diameter and elevated MHI values exhibit significantly greater intra-observer and inter-observer variability in the assessment of both LVEF and LV-GLS compared with subjects with normal thoracic conformation [28]. These findings suggest that chest wall geometry may influence not only cardiac dimensions and myocardial deformation itself, but also the reproducibility of the echocardiographic measurements used to evaluate cardiac function. The mechanical effects of thoracic constriction, including altered cardiac positioning, limited acoustic windows, and non-uniform ventricular motion secondary to external compression, may contribute to the increased variability observed in individuals with narrow thoraces.

From a clinical perspective, thoracic morphometry may therefore provide an additional framework for interpreting echocardiographic findings, particularly when repeated measurements show unexpected discrepancies or when myocardial deformation parameters appear discordant with the overall clinical picture. Assessment of thoracic conformation may help identify individuals in whom measurement variability is partly attributable to anatomical factors rather than to true changes in cardiac function.

The availability of a dedicated patented anatomical measurement device may further facilitate the routine incorporation of thoracic morphometry into cardiovascular imaging laboratories by providing a rapid, standardized, and operator-independent assessment of thoracic dimensions.

Future prospective studies will be necessary to determine whether systematic integration of thoracic morphometric assessment can improve the interpretation, reproducibility, and clinical applicability of conventional and advanced echocardiographic parameters.

## 5. Future Bioengineering Perspectives

The development of a dedicated patented anatomical measurement device represents only the first step in the technological evolution of thoracic morphometry. Although the current system was conceived as a portable mechanical device for direct anatomical measurement, its modular architecture provides a foundation for future technological advancements and digital integration.

Future generations of thoracic measurement systems may incorporate electronic distance sensors, digital readout displays, wireless communication modules, and automated calculation algorithms capable of providing real-time determination of the MHI. Integration with dedicated mobile applications and cloud-based platforms could facilitate standardized data acquisition, centralized data storage, and seamless integration of thoracic morphometric information into cardiovascular and respiratory clinical workflows.

The increasing availability of portable imaging technologies also creates opportunities for integration between thoracic morphometry and advanced cardiovascular imaging. Future devices may incorporate three-dimensional surface scanning systems, optical sensors, ultrasound-based distance measurement technologies, and multimodal imaging platforms capable of combining thoracic morphometric data with echocardiographic, electrocardiographic, and respiratory functional parameters. Such innovations may improve measurement accuracy, reduce operator dependency, and facilitate large-scale implementation in both clinical practice and epidemiological research.

In addition, artificial intelligence (AI) and machine learning algorithms may enable automated thoracic phenotype classification, identification of previously unrecognized morphometric patterns, and prediction of disease-specific cardiovascular and respiratory outcomes. By integrating thoracic morphometry with echocardiographic, pulmonary function, electrocardiographic, and clinical data, future AI-based models may improve cardiovascular phenotyping, refine risk stratification, and facilitate longitudinal monitoring of disease progression and treatment response rather than changes in thoracic morphology itself.

These developments may ultimately facilitate the transition of thoracic morphometry from a niche anatomical measurement to a broadly applicable tool for cardiovascular and respiratory phenotyping, personalized risk stratification, and precision medicine. In this context, the concept of digital thoracic phenotyping may emerge as a novel paradigm aimed at characterizing how thoracic geometry influences cardiovascular and respiratory structure, function, imaging findings, and clinical outcomes through standardized, reproducible, and non-invasive assessment of chest wall morphology.

## 6. Methodological Considerations and Validation Challenges

Several limitations should be acknowledged when interpreting the current evidence regarding thoracic morphometry, the MHI, and the novel patented anatomical measurement device.

The available literature on thoracic morphometry remains relatively limited and originates predominantly from a single research group and clinical institution. Although multiple studies have consistently demonstrated significant associations between thoracic conformation and cardiovascular or respiratory parameters, independent validation across different centers and operators is still lacking. To address this issue, a prospective multicenter study comparing the original MHI with the conventional radiological HI has recently been approved and is expected to provide the first formal external validation of the methodology.

Another important consideration is that most available investigations have included relatively small and highly selected populations, such as individuals with PE, MVP, obesity, pregnancy, pulmonary diseases, or AF. Moreover, the observational design of the majority of studies precludes definitive conclusions regarding causality. Whether thoracic morphology directly contributes to disease development or primarily acts as a modifier of cardiopulmonary phenotype remains incompletely understood.

The patented anatomical measurement device described in this review was specifically developed to improve standardization, portability, and reproducibility of thoracic morphometric assessment. However, despite patent protection and the strong engineering rationale supporting its design, the device has not yet undergone formal clinical validation. Consequently, its measurement accuracy, reproducibility, inter-observer variability, and agreement with both the conventional radiological HI and the original MHI remain to be established through dedicated prospective studies.

A further limitation relates to the intrinsic methodological differences between the original MHI and the measurement principle adopted by the new device. In the original MHI methodology, the A–P diameter is obtained echocardiographically as the distance between the ultrasound entry point at the skin surface and the posterior wall of the descending aorta, which serves as a surrogate marker of the anterior surface of the vertebral body. This approach essentially excludes the thickness of the anterior and posterior thoracic soft tissues. In contrast, the patented device directly measures the external A–P thoracic diameter between the sternal reference point and the vertebral reference element, thereby incorporating the contribution of thoracic soft tissues into the measurement. Although both approaches are intended to characterize thoracic morphology, this methodological difference may generate systematic discrepancies between the two techniques and should be carefully evaluated before they can be considered interchangeable. An additional limitation concerns the measurement of the L–L thoracic diameter. Unlike the conventional radiological HI, which is based on the internal transverse thoracic diameter measured between the inner margins of the rib cage, the MHI uses the maximum external L–L thoracic diameter, thereby incorporating the overlying soft tissues, including subcutaneous tissue and skeletal muscle. Consequently, individuals with marked thoracic musculature or increased thoracic soft tissue thickness may exhibit higher MHI values that do not exclusively reflect the underlying thoracic skeletal geometry, potentially leading to overestimation of thoracic narrowing and misclassification of thoracic morphology. Future studies should therefore investigate whether correction strategies or body habitus-adjusted reference values could further improve the accuracy and generalizability of MHI measurements across different populations.

At the same time, this review possesses several strengths. It provides, to our knowledge, the first comprehensive overview integrating the historical evolution of thoracic morphometry, the development and clinical applications of the MHI, and the engineering rationale underlying a dedicated patented measurement system. By combining clinical, imaging, and bioengineering perspectives, the review proposes a broader conceptual framework in which thoracic morphology is considered not merely an anatomical descriptor but a potential determinant of cardiopulmonary phenotype and an emerging component of precision cardiovascular and respiratory medicine.

## 7. Conclusions

Thoracic morphometry has progressively evolved from a tool primarily used for the assessment of PE to a broader approach for cardiovascular and respiratory phenotyping. The development of the MHI represented a major step toward radiation-free evaluation of chest wall geometry and enabled the investigation of thoracic conformation across multiple clinical settings.

Accumulating evidence indicates that chest wall morphology influences cardiac chamber geometry, myocardial deformation parameters, ESE findings, respiratory physiology, and clinical outcomes in several cardiovascular and pulmonary diseases. These observations support the concept that thoracic conformation represents an important but often overlooked determinant of cardiopulmonary phenotype.

The development of a novel patented anatomical measurement device specifically designed for thoracic morphometric assessment represents a further technological advance aimed at improving measurement standardization, reproducibility, portability, and clinical usability. Future integration with digital technologies, artificial intelligence, and advanced imaging platforms may further expand the role of thoracic morphometry in precision medicine.

Although additional multicenter validation studies are required, current evidence suggests that thoracic shape assessment may become an increasingly valuable component of contemporary cardiovascular and respiratory evaluation. The convergence of clinical research, biomedical engineering, and technological innovation offers exciting opportunities for the future development of personalized thoracic phenotyping and non-invasive cardiopulmonary assessment.

## Figures and Tables

**Table 1:** Comparison between the conventional radiological Haller Index (HI) and the Modified Haller Index (MHI). Validation data are based on the original prospective study of 100 consecutive subjects, which demonstrated excellent agreement between the two methods, supporting the validity of the MHI as a reliable radiation-free alternative to the conventional radiological HI.

Characteristic	Conventional Haller Index (HI)	Modified Haller Index (MHI)
Measurement principle	Internal thoracic dimensions	Combined external anthropometric and echocardiographic measurements
Latero-lateral diameter	Internal transverse chest diameter measured by CT or chest radiography	Maximum external transverse thoracic diameter measured directly on the chest wall
Antero-posterior diameter	Internal sterno-vertebral distance measured by CT or chest radiography	Distance between the most depressed point of the sternum and the anterior surface of the vertebral body
Radiation exposure	Yes	No
Imaging equipment required	CT scan or chest radiography	Graduated anthropometric caliper and conventional transthoracic echocardiography
Bedside applicability	Limited	Yes
Repeatability for serial follow-up	Limited by radiation exposure	Unlimited
Time required	Imaging acquisition and post-processing	Few minutes
Cost	Moderate to high	Low
Accessibility	Requires radiology facilities	Applicable in outpatient and bedside settings
Correlation with radiological HI	Reference standard	r = 0.81 (*p* < 0.001)
Mean value in validation study	1.93 ± 0.35	1.99 ± 0.30
Main advantage	Established reference standard	Radiation-free, portable, rapid, and repeatable
Main limitation	Radiation exposure and resource utilization	Requires dedicated measurement procedure

## Data Availability

The dataset generated from the extraction of data from the included studies will be made openly accessible through Zenodo (https://zenodo.org; accessed on 24 June 2026).
